# How transform fault shear influences where detachment faults form near mid-ocean ridges

**DOI:** 10.1038/s41598-023-35714-3

**Published:** 2023-06-07

**Authors:** Jana C. Schierjott, Garrett Ito, Mark D. Behn, Xiaochuan Tian, Thomas Morrow, Boris J. P. Kaus, Javier Escartín

**Affiliations:** 1 School of Ocean and Earth Science and Technology, University of Hawai’i, Honolulu, USA; 2 Department of Earth & Environmental Sciences, Boston College, Chestnut Hill, MA USA; 3 NOAA Ocean Exploration, Silver Spring, MD USA; 4 Institute of Geosciences, Johannes Gutenberg University, Mainz, Germany; 5 Laboratoire de Géologie, CNRS UMR 8538, ENS, PSL University, Paris, France

**Keywords:** Tectonics, Geodynamics, Structural geology

## Abstract

Oceanic detachment faults represent an end-member form of seafloor creation, associated with relatively weak magmatism at slow-spreading mid-ocean ridges. We use 3-D numerical models to investigate the underlying mechanisms for why detachment faults predominantly form on the transform side (inside corner) of a ridge-transform intersection as opposed to the fracture zone side (outside corner). One hypothesis for this behavior is that the slipping, and hence weaker, transform fault allows for the detachment fault to form on the inside corner, and a stronger fracture zone prevents the detachment fault from forming on the outside corner. However, the results of our numerical models, which simulate different frictional strengths in the transform and fracture zone, do not support the first hypothesis. Instead, the model results, combined with evidence from rock physics experiments, suggest that shear-stress on transform fault generates excess lithospheric tension that promotes detachment faulting on the inside corner.

## Introduction

Detachment faults are long-lived (1–3 Myr^1^), low-angle normal faults ($\le$ 20–30$^{\circ }$^2–4^) that were first discovered at continental rifts, such as the Basin-and-Range, e.g. refs.^5–8^. They accumulate extremely large offsets, leading to exhumation of middle-lower crust and/or mantle^5,9^ that is often metamorphosed in continental settings. In the oceanic realm, detachment faults represent an end-member form of normal faulting and seafloor creation^10–13^, with the exposed surface of the hanging wall often forming a topographic high, known as oceanic core-complex^1,14–16^.

**Figure 1:**
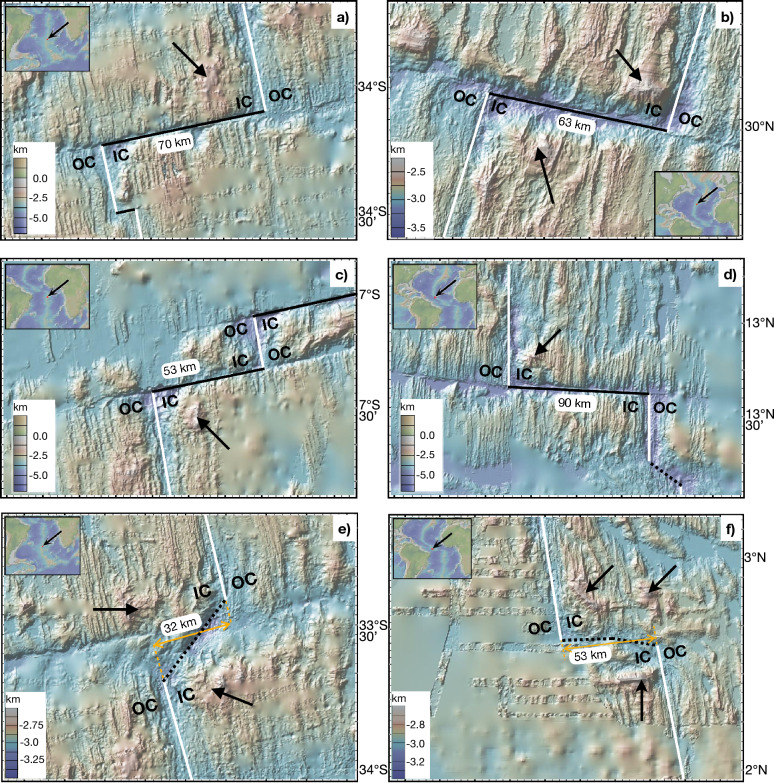
Bathymetry maps showing examples of detachment faults on inside corners at the intersections between seafloor spreading ridges (white lines) and transform faults (black lines) (**a**)–(**d**), and non-transform offsets (dotted lines) (**e**) and (**f**). All inside corner and outside corners are marked with IC and OC, respectively. Black arrows point to detachment faults. Spreading-parallel length of transform or non-transform offsets are labeled (distances in km). Bathymetry maps are made with GeoMapApp (https://www.geomapapp.org/)/CC BY^29^.

Detachment faults are found primarily at slow to ultra-slow spreading ridges^3,11^ and are linked to reduced magmatic supply^1,17,18^. Observations^11,14,17^ and numerical modeling^9,19–21^ indicate that detachment faults occur when the amount of seafloor spreading accommodated by magmatic intrusions is approximately 50$\%$. This is consistent with the frequent occurrence of detachment faults near the ends of slow spreading ridge segments where magmatic crustal thickness is reduced relative to segment centers^22–26^. When adjacent to segment offsets, detachments faults primarily occur at the inside corner (IC), or transform fault-side, of the ridge-transform intersection^3,11^ as opposed to the outside corner (OC), or fracture zone-side, of the ridge-transform intersection, showing a systematic asymmetry (Fig. 1) indicating that magma supply alone is not the only process affecting their formation. This tendency of detachment faults to occur on the inside corner of ridge-transform intersections is poorly understood and few studies have quantitatively investigated the causes for the asymmetry in detachment faults and their associated topography. For example, while two-dimensional (2-D) modeling studies have demonstrated the importance of magmatic intrusions that partially accommodate seafloor spreading to the spacing, size, and longevity of normal faulting at mid-ocean ridges^9,20,27^, these studies do not predict the cause of this asymmetry. Moreover, the few three-dimensional (3-D) modeling studies that have examined the role of along-axis variations of magma intrusions in the lithosphere on the stability and along-axis extent of detachment faults, have done so only in the absence of a transform fault^19,28^.

Even before detachment faults were recognized as the cause for inside corner topographic highs, ref.^30^ hypothesized that they may result from the contrast in plate-coupling between the transform fault (TF) and the fracture zone (FZ), supporting a “decoupling” hypothesis put forward by previous authors, e.g. refs.^31,32^. According to ref.^30^ the slipping transform fault weakens the plate boundary, mechanically decoupling the plate such that the inside corner can rise in response to the forces that uplift transform valley walls. By contrast, the inactive fracture zone forms a stronger weld with the adjacent plate, which inhibits uplift. This conception model^30^ is consistent with numerical simulations that indicate transform faults are weak and support little mechanical coupling across the fault^33^. These concepts lead us to hypothesize that the differences in the amount of coupling across the transform fault (weaker) and fracture zone (stronger), promote detachment faulting and enhanced uplift on the decoupled inside corner (hypothesis 1). A second hypothesis arises from the initial set of numerical experiments conducted in this study, which are presented below. This alternative hypothesis states that shear stress on the transform fault causes elevated horizontal tension in the adjacent lithosphere, which promotes detachment faulting preferentially on the inside corner.

In order to investigate the cause for detachment faulting on the inside corners of ridge-transform intersections, we employ 3-D numerical models that simulate the combination of faulting in the brittle lithosphere, a ridge-transform-ridge geometry, and intrusions that partially accommodate seafloor spreading. In the base model, magmatic intrusions accommodate a fraction of 75$\%$ of seafloor spreading, with the remaining fraction of 25$\%$ accommodated by normal faulting. These models predict relatively short-lived, abyssal hill-forming normal faults on both sides of the ridge, even near the transform faults. In contrast, models where magmatic intrusions accommodate only 50-60$\%$ of total seafloor spreading predict long-lived detachment faults (>1.0 Myr) that form preferentially on the inside corners. We systematically vary the frictional strengths of the transform fault and fracture zone to test the two above mentioned hypotheses with regard to the observed asymmetry in detachment faulting near ridge-transform intersections.

## Modelling approach and results

**Figure 2:**
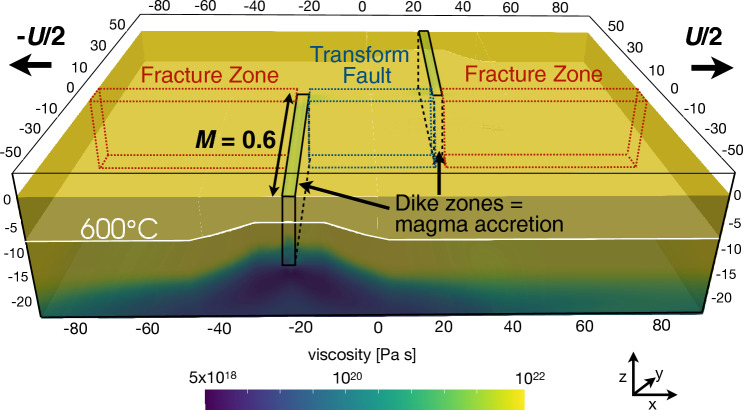
This image of the model set up shows the two ridge segments, where mass is injected in dike zones to drive magmatic extension at a fraction *M* times the spreading rate. White contour shows the 600 °C isotherm of the initial temperature, colors show corresponding effective (visco-elasto-plastic) viscosity. Red and blue outlines schematically show the fracture zones and transform fault areas (all 15 km deep^9,38^), respectively, in which the friction angle is altered. All distances are in km.

The 3-D numerical experiments modeled with the code LaMEM^34^ simulate two mid-ocean ridge segments separated by a 30 km-long transform fault which can form spontaneously and evolve dynamically (see “Methods”). Natural detachment faults are observed at full spreading rates (<75 km/Myr, e.g., ref.^21^), being more common at slow^35^ spreading rates (<55 km/Myr). We therefore choose a representative full spreading rate of 40 km/Myr^36,37^ which is imposed on the two opposite sides of the model domain (Fig. 2). Along the ridge segments dike injection is simulated by imposing the amount of seafloor spreading accommodated by magmatic intrusions (*M*). If *M* = 0.6, for example, the dike zones open at a rate of 60$\%$ of the full spreading rate, and the remaining plate spreading must occur tectonically by normal faulting. In this study, we examine models in which *M* is uniform at 0.75, 0.6, and 0.5, as well as a model in which *M* decreases along the ridge segments from 0.8 far from the transform zone to 0.6 at the ridge-transform intersection.

### Abyssal hill faults

**Figure 3:**
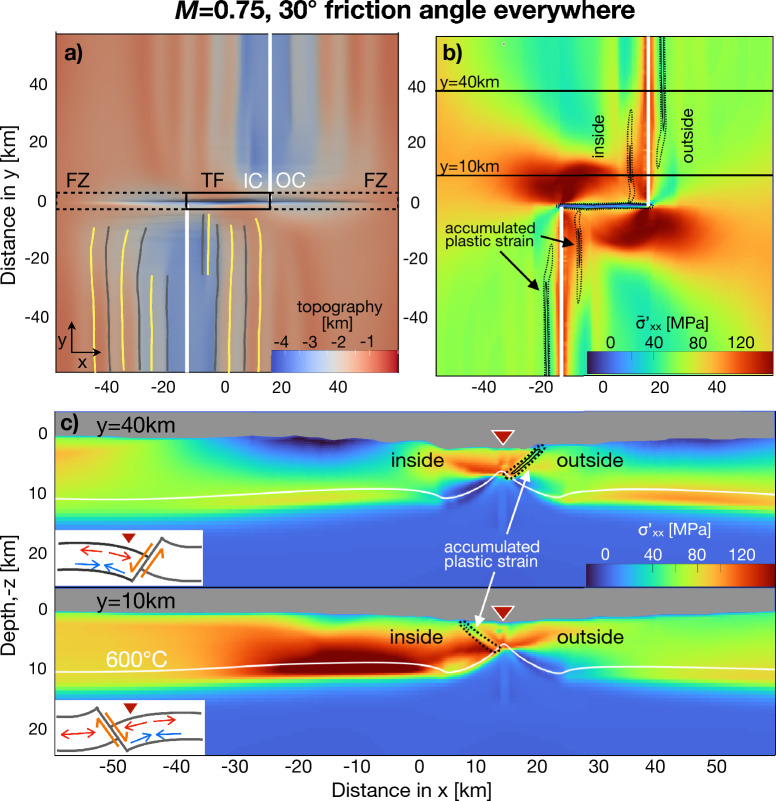
Model results for base model with *M* = 0.75 are shown. For visualization, the images are restricted to +/–60 km in *x* and *y*. (**a**) Map view of model topography at 2.0 Myr, showing transient faulting on both sides of the ridge axis creating abyssal hill-like topography. Black solid and dashed rectangles outline the transform fault and fracture zones, respectively. White lines mark dike injection zones. Yellow lines mark fault breakaways (i.e., where the fault scarp was first exposed) and grey lines mark fault terminations. A pair of inside and outside corners is marked with IC and OC, respectively. (**b**) Map view of deviatoric horizontal normal stress ($\sigma ^{'}_{xx}$, positive for tension) averaged in depth between the seafloor and the 600 °C-isotherm. Black solid and black dotted contours show accumulated plastic strain of 0.2 and 1.2, respectively, revealing active faulting on the fracture zone-side far from the transform fault and on the inside corner near the transform fault. $\sigma ^{'}_{xx}$ is tensile along the ridge segments and most tensile in a broad area on either side of the transform fault. (**c**) Vertical cross-sections of $\sigma ^{'}_{xx}$ at *y* = 40 km and *y* = 10 km (located by black lines in panel **b**). Red triangles locate the dike zone, black contours are as in (**b**), and white line marks the 600 °C-isotherm. At a distance of *y* = 40 km from the transform fault (**c** top) downward bending of the hanging wall lithosphere causes $\sigma ^{'}_{xx}$ to be tensile near the top and compressive near the bottom of the lithosphere. Close to the transform fault (*y* = 10 km, **c** bottom), $\sigma ^{'}_{xx}$ is generally more tensile in the deeper part of the lithosphere attached to the footwall than in the hanging wall.

To understand why detachment faults preferentially develop on one side of the ridge axis, we must first gain insight into the more typical situation of faulting that occurs on both sides of the ridge axis. We run a base case with *M* = 0.75 and a friction angle of $30^\circ$ (coefficient of friction is 0.6) everywhere including along the transform fault and fracture zone. Normal fault-bounded rift valleys form along each ridge segment (Fig. 3a), which is consistent with observations of abyssal hills along slow-spreading ridges in nature^39,40^. The model also predicts a valley along the transform fault where left-lateral simple shear localizes. The width of the transform fault valley is controlled by the imposed geometry of the dike zones (dike zone on the north segment ends at the same *y*-location where the dike zone on the south segment begins). Parallel to and flanking the transform valley, ridges of elevated topography are visible, sometimes superimposed with linear discontinuities. These artifacts arise because the added source term in the continuity equation (see “Methods”) for the dike zone, does not control which direction the dike zone expands. Shear stress near the TF inhibits horizontal expansion, and promotes vertical expansion, which generates the topography. As the elevated topography occurs on both sides of the ridge segments, it does not influence on which side faulting occurs and hence does not impact our main results.

In this base model, normal faulting occurs on both sides of the ridge segments forming seafloor fabric resembling abyssal hills (Fig. 3a, b). During the time step shown, along one third of the segment, an active normal fault is located on the inside (transform fault-side) of the ridge segment. Along the rest of the segment, an active fault is located on the outside (fracture zone-side) of the ridge segment (Fig. 3b). The pattern of deviatoric horizontal normal stress, $\sigma ^{'}_{xx}$, shown in a vertical cross-section perpendicular to the ridge axis far from the transform fault (*y* = 40 km), provides insight as to why faults alternate sides of the ridge axis (Fig. 3c). While the fault is slipping, the lithosphere attached to the hanging wall bends downward in response to the downward slip along the fault^41^. This bending leads to elevated tensile $\sigma ^{'}_{xx}$ in the upper part and relative compressive $\sigma ^{'}_{xx}$ in the deeper part of the lithosphere (compare to schematic sketch in Fig. 3c. Eventually, the tensile $\sigma ^{'}_{xx}$ in the upper part is sufficient to initiate a conjugate fault in the hanging wall lithosphere on the opposite side of the ridge axis. This process oscillates through time, explaining the alternation of normal faulting from one side of the ridge axis to the other (supplement S1, S5, S6). These model results also provide another perspective for understanding the cause of the prevalence of conjugate normal faults (graben formation) in many extensional environments^42^. Normal faulting on both sides of a mid-ocean ridge and graben formation at rift zones may share a basic physical origin.

The effects of the transform fault on $\sigma ^{'}_{xx}$ are evident in map view (Fig. 3b). There is a large zone of excess tension on either side of the transform fault (Fig. 3c, bottom, horizontal normal stress is shown as the average between the seafloor and the 600 °C-isotherm, tension-positive sign convention). This elevated horizontal tension arises from the shear stress on the transform fault; the tension acts to pull the two plates laterally past one another against the frictional resistance of the transform fault. The transform fault-induced excess tension promotes continued faulting on the inside corner, but evidently, in this model with *M* = 0.75, the asymmetry in $\sigma ^{'}_{xx}$ is insufficient to keep the fault on the inside corner and the faulting switches between the inside and outside along the entire lengths of the ridge segments, including close to the transform fault. However, each fault on the inside corner remains active for a greater duration (typically 0.4–0.5 Myr) than each outside corner-fault ($\approx 0.05$ Myr). At greater distance from the transform fault, where the excess tension due to the transform fault is absent, the normal faults typically stay active for 2.0 Myr on both sides of the ridge segment (similar to 2-D model results with only a ridge and no TF, Fig. S1 in supplement). According to these findings, the elevated tensile stress on the inside corner promotes relatively long-lived faulting on the inside compared to the outside corner, but is insufficient to completely prevent normal faulting on the outside corner.

### Detachment faults on inside corners

**Figure 4:**
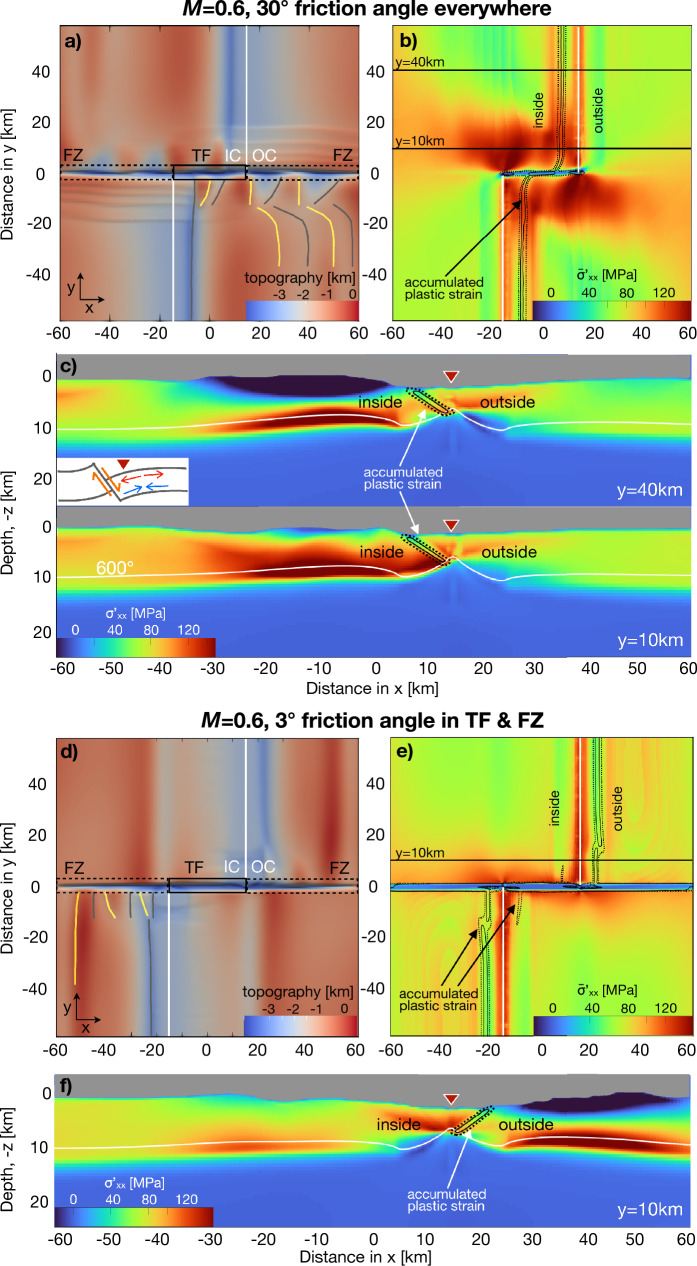
Results for two different models with *M* = 0.6. Symbols are as defined in Fig. 3. (**a**)–(**c**) Model with the base friction angle of $30^\circ$ everywhere. (**a**) Map of model topography at 3.0 Myr. The fault is located on the inside. Close to the transform fault, semi-permanent detachment faults lead to recurring inside corner-highs. (**b**) Plastic strain contours reveal faulting on the inside along the ridge segments. $\sigma ^{'}_{xx}$ shows similar patterns and has similar causes (bending and shear on the TF) as the previous model with *M* = 0.75. (**c**) Far from the transform fault (*y* = 40 km, top), downward bending of the hanging wall lithosphere causes $\sigma ^{'}_{xx}$ to be tensile near the top and compressive near the bottom of the lithosphere. Close to the TF (*y* = 10 km, bottom), $\sigma ^{'}_{xx}$ is generally more tensile in the lithosphere on the inside corner and than on the outside corner. (**d**)–(**f**) Model with friction angle of $3^\circ$ in FZs and TF. (**d**) Map of model topography at 2.0 Myr. The fault is located on the outside. Recurring detachment faults form close to the FZs on the outside. (**e**) Tensile stresses are elevated along ridge segments and in a very small region very close to the TF. (**f**) Vertical cross-sections of $\sigma ^{'}_{xx}$ at 10 km from the TF. The stress field is very similar to (**c**) top, but reversed, left-to-right, indicating little or no perturbation of $\sigma ^{'}_{xx}$ due to the (very weak) shear stress on the TF.

Next, we evaluate a model with *M* = 0.6, a value similar to the $\sim 50\%$ associated with detachment faulting^11,21^. The model shows normal faulting to switch sides of the ridge axis in the beginning of model run ($t<$1.5 Myr) (supplement S6), but eventually, a persistent detachment fault forms on the inside of each ridge segment (Fig. 4a, b). The topography further reveals that the part of the detachment fault close to the transform fault periodically jumps back to the ridge axis, thereby creating individual, separated inside corner highs. Simultaneously, at greater distance from the transform fault, incipient shear bands begin to localize on the fracture zone-side of the ridge axis, but never establish as a stable normal fault. A model with *M* = 0.5 produced a similar result, however, in that case there is no transient behavior of the detachment fault, it remains stable and on the inside corner for $>0.5$ Myr (supplement S2).

The stress pattern of the model with *M* = 0.6 and long-lived detachment faulting is qualitatively similar to that of the previous model with *M* = 0.75 and shorter-lived abyssal hill faults. Far from the transform fault, bending of the hanging wall results in tensile and compressive $\sigma ^{'}_{xx}$ in the upper and lower part of the lithosphere, respectively (Fig. 4c, top). Close to the transform fault, a large portion of the lower part of the footwall lithosphere is more tensile inside next to the transform fault than on the outside. The map view of depth-averaged $\sigma ^{'}_{xx}$ (Fig. 4b), likewise, shows a broad area of tensile stress on either side of the TF. The key difference in the *M* = 0.6 model compared to the base case with *M* = 0.75, is that the elevated $\sigma ^{'}_{xx}$ around the transform fault is sufficient to keep the faulting on the inside corner, given the conditions (*M* = 0.6) favorable for forming a detachment fault.

The above results lead to the second hypothesis for the origin of inside corner-detachment faults stated earlier: shear stress on the transform fault leads to elevated horizontal tension and therefore generally promoting faulting on the inside corner. For conditions which favor detachment faulting ($M\approx 0.5$), the elevated horizontal tension keeps the fault on the inside corner.

### Detachment faults on outside corners

We next examine a model (*M* = 0.6) in which both the transform fault and fracture zone are weakened by an imposed friction angle of 3$^\circ$ inside those regions, compared to the 30$^\circ$ in the rest of the lithosphere (Fig. 4d–f). Initially ($t<$2.0 Myr), this model predicts a single fault along each ridge segment which switches between the inside and outside. Eventually, the fault stabilizes as a long-lived detachment fault on the outside corner. Although this behavior has not been documented in nature, the results provide insight as to why outside corner-detachment faults do not form, which helps in understanding the general, governing physics of detachment faults.

The pattern of $\sigma ^{'}_{xx}$ is different from that of the prior model with *M* = 0.6 and the reference friction angle of 30$^\circ$. In map view, depth-averaged $\sigma ^{'}_{xx}$ is largely symmetric on either side of the ridge axis, and is elevated on the inside corner in only a small zone close to the transform fault (Fig. 4e, supplement S5). Also, the vertical cross-section close to the transform fault displays relatively little excess horizontal tension on the inside corner (Fig. 4f). In fact, the magnitude and pattern of $\sigma ^{'}_{xx}$ on the hanging wall close to the transform fault, which here extends to the inside corner, are very similar to those of the outside hanging wall far away from the transform fault in the prior model with the reference friction angle (compare Fig. 4f and c, top). Thus, it appears that the degree of asymmetry in $\sigma ^{'}_{xx}$ between the inside and outside corner is important to determine where detachment faulting occurs.

## The cause for inside corner-detachment faults

Here, we analyse a wide range of numerical models in light of the two previously mentioned hypotheses on the cause for inside corner-detachment faults. Again, hypothesis 1 posits that the lower coupling across the transform fault promotes inside corner-detachment faults, whereas hypothesis 2, inspired by our numerical model results, states that the asymmetry in stress is responsible for inside corner-detachment faults. We explore solutions for a range of friction angles along the transform fault ($\varphi _{TF}$) and fracture zone ($\varphi _{FZ}$). Key properties to consider are the relative strengths of the transform fault ($S_{TF}$) and fracture zones ($S_{FZ}$). For this we show the results in terms of shear stress, integrated over the approximate thickness of the lithosphere (*H* = 7 km), assuming the normal stress is lithostatic. These calculations take into account that the active transform fault has lost almost all cohesion ($C_{TF}$ = 2 MPa, but still has frictional strength), whereas the fracture zone has the full cohesion $C_{FZ}$ of 40 MPa (supplement S3).

**Figure 5:**
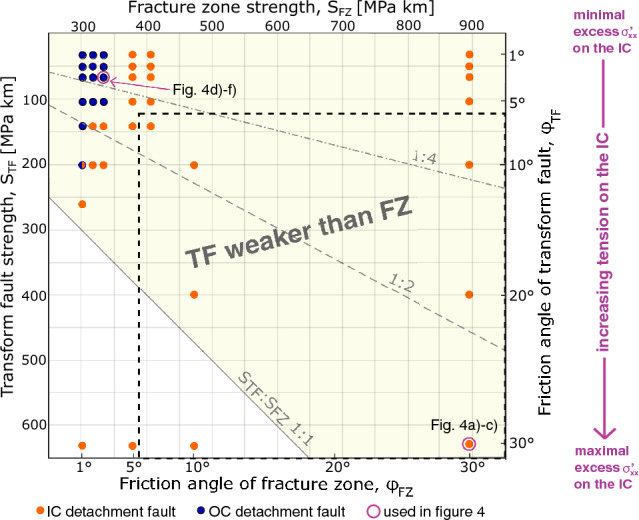
Regime diagram depicting all models with *M* = 0.6 and full spreading rate of 40 km/Myr and 30 km length, evaluated for their detachment fault location. Orange dots indicate detachment faults on the inside corner, blue dots indicate detachment faults on the outside corner. The orange-blue dot depicts a model in which the detachment fault does neither localize on the in- nor on the outside (runtime of 4.0 Myr). The horizontal and vertical axes are estimated strengths of the transform fault and fracture zone, respectively, based on the integrated shear strengths (see “Methods”). The TF is weaker than the FZ in the yellow-shaded area. Lines are delineate strength ratios of TF to FZ of 1:1, 1:2 and 1:4. Corresponding friction angles are labeled on the bottom and right axes. The values predicted by rock physics experiments fall into the area outlined by the dashed box. Models with longer transform faults (40 km and 50 km) show very similar results. Indeed, those model results predict inside corner-detachment faults for even more cases (see supplement S7).

Under hypothesis 1, decoupling on the TF would be promoted with lower transform fault-strength relative to that of the fracture zone. Therefore inside corner-detachment faults would be more favored as the transform fault becomes weaker compared to the fracture zone. However, the approximate opposite is observed: outside corner faults occur at very low ratios of transform fault- to fracture zone-strength, and inside corner faults occur in the majority of cases, including high ratios of transform fault-to fracture zone-strength (Fig. 5). Still, hypothesis 1 can not yet be rejected because at the lowest transform fault strengths ($\approx$100 MPa and less), the transition between outside- and inside corner detachments coincides with increasing facture zone strength, suggesting the contrast in strength with the tranform fault is important when both strengths are extremely low (upper part of Fig. 5).

Hypothesis 2, which predicts that greater transform fault-strength leads to greater excess $\sigma ^{'}_{xx}$ near the transform fault promoting inside corner-detachment faults, is broadly supported by our model results. We find that outside corner-detachment faults only occur when the transform fault is very weak and hence, little or no excess $\sigma ^{'}_{xx}$ can accumulate (Fig. 5). However, for a small subset of models, when the transform fault is very weak but the fracture zones are not, the models predict inside corner-detachment faults even though outside corner-detachment faults would be expected due to the weakness of the transform fault (Fig. 5, upper right quadrant). A simple interpretation is that even if a weak transform fault and the associated lack in excess $\sigma ^{'}_{xx}$ should allow for an outside corner-detachment fault, a competent fracture zone prevents this from happening.

Even though we find broad support for hypothesis 2, we cannot completely rule out hypothesis 1 as the cause for inside corner-detachment faults. Thus, we take into account both geological evidence and laboratory experimental work to distinguish the two hypotheses further. For this, we consider serpentinite and talc, which are rocks found in transform faults and are known to be frictionally weak. These rocks, in addition to other hydrous rocks, result from faults and cracks that are linked to tectonic deformation around oceanic faults in general, and transform faults in particular. Cracks facilitate fluid flow and syntectonic fluid-rock interaction (e.g., refs.^43–46^). Furthermore, seismic studies suggest that transform faults might contain a non-neglectable amount of serpentinite attributed to their relatively low seismic coupling and mode of fault slip^47,48^.

Various studies of laboratory experiments have been performed to estimate the frictional strength of talc and serpentinite^45,49–53^ collected from fault gouge material, exhumed ophiolites, and dredged from the seafloor. The smallest experimentally estimated friction coefficient presented is $\mu =0.1$ ($\varphi =5.7^\circ$) for the low-temperature serpentine polymorph chrysotile^51^, as well as for talc-serpentine mixtures^54^ (in both studies experiments were performed under water-saturated conditions). Other estimates for friction coefficients are higher. For example, published estimates for lizardite are $\mu =0.15$–0.19 ($\varphi =8.5$–10.7°)^53^, $\mu =0.15$–0.35 ($\varphi =8.5$–19.3°)^52^, and $\mu \approx 0.5$–0.56 ($\varphi \approx 26.6$–29.3°)^55^. Antigorite tends to have an overall higher friction coefficient: $\mu \approx 0.50$–0.85 ($\varphi \approx 26.6$–40.4°)^52,53,55^.

The presented values of frictional strengths place rocks which are typical for transform faults and fracture zones in the lower right portion of the regime diagram in Fig. 5. The same area of the diagram is where the models predict significant excess $\sigma ^{'}_{xx}$ and detachment faulting on the inside corner (hypothesis 2). The extremely weak transform faults required by hypothesis 1 are unlikely given the above experimental results and when considering that the majority of the volume of rock that is slipping in the TF would have to be serpentinized. Hence, hypothesis 2 provides the better explanation for the preference of detachment faults to form on inside corners.

**Figure 6:**
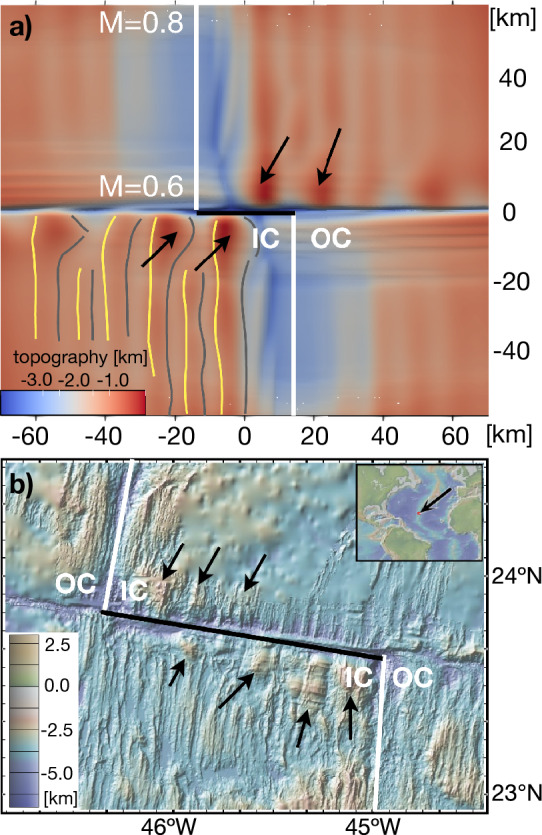
Comparison of a more realistic model result with natural observations. (**a**) Topography of a model with tapering *M* along the ridge axis from 0.8 at the model boundary to 0.6 at the transform fault. Symbols are as defined in Fig. 3. Semi-permanent detachment faults lead to recurring topographic highs at the inside corners. Away from the transform fault, abyssal hills are located on either side of the ridge axes where faulting occurs on both sides of the ridge segments. (**b**) Bathymetry map of a ridge-transform intersection of the Mid-Atlantic ridge at 23–24$^\circ$ North and 45–46$^\circ$ West. Abyssal hills along both ridge segments and prominent, discrete topographic highs along the tranform fault (black arrows) are visible. Bathymetry map is made with GeoMapApp (https://www.geomapapp.org/)/CC BY^29^.

Finally, to validate our numerical models, we compare the outcome of a more realistic numerical simulation to natural observations. So far, the discussed simulations represented idealized ridge-transform intersections in which *M* is imposed to be uniform along the entire lengths of the ridge segments. However, given that magma supply at slow-spreading ridges often decreases towards transform faults, e.g. refs.^22,24–26^, we present a model in which *M* decreases from 0.8 at the edges of the model, representing the centers of ridge segments, to 0.6 at the ridge-transform intersection (supplement S4). Similar models, but using only a single ridge segment without a transform fault, have been performed before^19^. The model topography shows qualitative similarities with the observed topography near the Mid-Atlantic Ridge (MAR) (Fig. 6b). The predicted seafloor topography reveals recurring, transient detachment faults (0.7–1.0 Myr lifetime) on the inside corner of the ridge-transform intersection (Fig. 6a). At a larger distance from the offset, the faults switch sides and lead to typical abyssal hills, similar to predictions by ref.^19^, and resembling the observed structure on the MAR. Using this set of model parameters, we are able to explain a variety of natural observations, including the transition from transient detachment faults to abyssal hills along one single ridge segment. Furthermore, the model shows that discrete inside corner highs do not rely on episodes of magmatic and amagmatic spreading, but instead can occur as transient behavior when *M* is slightly larger than 0.5 (recall, M = 0.5 leads to stable detachment faults according to e.g. refs.^9,19,28^, see also supplement S2).

In this study, we investigate the origin of the observed asymmetric occurrence of detachment faults on the inside corners of ridge-transform intersections. Our study shows that the cause is elevated tension on the inside corner associated with shear stress on the TF, not a decoupling effect of the TF as previously proposed. Indeed, it may be that a similar pattern of excess tension promotes detachment faults to form on the inside corner of non-transform offsets as well; however, future studies are needed to test this, given the variable and complex geometry and structure of different non-transform offsets. Broadly speaking, this study shows the importance of magmatic and tectonic interaction in forming a range of seafloor fabrics at slow-spreading mid-ocean ridges, including the end-member form of seafloor creation formed by detachment faults and oceanic core complexes^10–13^.

## Methods

The numerical code LaMEM^34^ employs a finite difference marker-in-cell technique to solve the mass, momentum and energy conservation equations on a fully staggered grid. In our models, the Cartesian model domain has dimensions of 178.0$\times$118.2$\times$28.4 km in *x*, *y*, *z*, respectively, using a variable grid consisting of 384$\times$192$\times$64 cells. The highest resolution, represented by a minimum grid size of 0.333$\times$0.4$\times$0.333 km, is within 30 km of each ridge segment in the spreading direction (*x*), within 20 km of the transform in the *y*-direction, and down to a depth of 11 km below the model seafloor.

The model has two layers: a 4 km-thick ocean layer overlying a lithosphere-asthenosphere layer 24.4 km in thickness. The ocean is simulated as a fluid of relatively low density (1000 kg/m^3^) and viscosity (5$\times 10^{18}$ Pa s) so as to simulate a quasi-stress-free lithospheric surface following the “sticky-air” approach^56,57^. The rock layer has a composite visco-elasto-plastic rheology. The shear modulus is set to G = 40 GPa and viscous deformation follows the power-law, temperature-dependent rheology for dry olivine in dislocation creep of the study by ref.^58^. Plasticity simulates brittle deformation during fault sliding, and the localization of shear bands, using the Drucker-Prager yield criterion. For this, lithostatic (not dynamic) pressure controls the frictional resistance to sliding. The friction angle is constant (with a reference value of 30$^\circ$) whereas cohesion, *C*, is reduced as a function of the damage parameter, accumulated plastic strain, $\varepsilon _{ap}$, e.g. refs.^19,20,27,59^. When $\varepsilon _{ap}\le 0.01$, *C* = 40 MPa; from when $\varepsilon _{ap}$ = 0.01 to when $\varepsilon _{ap}$ = 0.15, *C* decreases linearly from 40 MPa to 2 MPa (i.e., $5\%$ of 40 MPa). For $\varepsilon _{ap}\ge$ 0.15, *C* = 2 MPa. Healing of damage is simulated following the approach by refs.^20,27^. At each model timestep, *dt*, $\varepsilon _{ap}$ is reduced according to the healing timescale $t_{heal}$ following $\varepsilon _{ap} = \frac{\varepsilon _{ap}}{\left( \frac{dt}{t_{heal}} +1.0\right) }$. The healing time is set to $t_{heal}=0.03$  Myr. Lower and upper cut-offs of $5\times 10^{18}$  Pa s and $5\times 10^{22}$ Pa s for effective (ductile+elastic+plastic) viscosity provide a sufficient dynamic range of rheologic behavior, while also optimizing computational speed and solution accuracy. Global mass conservation is ensured with an imposed inflow of asthenosphere at the bottom of the model domain, and a free in and out flow condition for ocean at the top of the domain.

Zones of dike injection are imposed at the centers of the ridge segments through the continuity equation^9,19,20,27,38^. Specifically, a mass source term is added to the right-hand-side to mimic the effect of magmatic intrusions, $\nabla \cdot \overrightarrow{\varvec{v}}= \frac{UM}{w_{d}}$. The source term depends on the dike zone width $w_{d}$, the full spreading rate *U*, and the spreading fraction accommodated magmatically *M*. If *M* = 0.6, for example, the dike zones open at a rate of 60$\%$ of the full spreading rate, and the remaining of plate spreading must occur tectonically by normal faulting. In the dike zone, the divergent part of the velocity field is not allowed to contribute to plastic strain and hence rheology, nor is it included in the momentum equation.

Temperature is controlled by thermal conduction, added heat by magmatism as well as hydrothermal circulation^20^. Cooling by hydrothermal circulation is simulated with thermal diffusivity that is enhanced by a factor of Nu = 5 within 10 km of each ridge segment down to the 600 °C-isotherm, similar to previous approaches such as refs.^20,60^. Temperature at the seafloor and base of the model is maintained at 1 °C and 1350 °C, respectively.

## Supplementary Information

Supplementary Information.

## Data Availability

The open-source code LaMEM by^34^ used for the numerical models in this study is available at https://bitbucket.org/bkaus/lamem/src/master/. All newly coded features are embedded in the master version of LaMEM. A “Readme” and input files for models shown in figures 3,4 and 6 in the main text as well as for figures in the supplement can be found at https://bitbucket.org/JanaNa/lamem/downloads/. A movie showing the evolution of the model in figure 6 can be found at the same address https://bitbucket.org/JanaNa/lamem/downloads/ and is described in the supplementary material.
