# Supplementary material for: How transform fault shear influences where detachment faults form near mid-ocean ridges
Source: Sci Rep. 2023 Jun 7;13:9259. doi: 10.1038/s41598-023-35714-3 (PMC10247753; doi:10.1038/s41598-023-35714-3)
Supplement: Supplementary file 1 — Supplementary Information. [file 41598_2023_35714_MOESM1_ESM.pdf]

Supplementary information for paper "How transform fault shear influences where detachment faults form near mid-ocean ridges"

S1: Figure of 2-D model with M=0.75

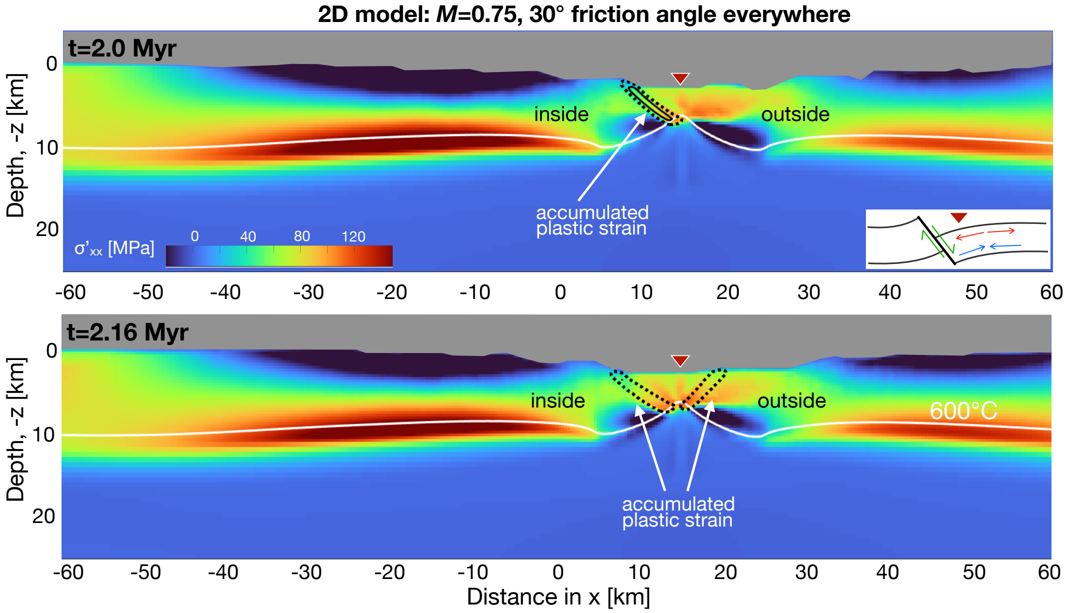

Supplementary Figure S 1. Result of a 2D-model with M=0.75 at two different time steps is shown. Except for the y-dimension, all parameters are the same in the 2D- and 3D-models (which are shown in the main text). Red triangle depict location of the ridge axis. Outlines show accumulated plastic strain of 0.2 (dashed line) and 1.2 (solid line), respectively. The top panel at t=2.0 Myr displays a localized fault on one side of the ridge axis. The bottom panel shows the same 2D-model at a later time step (t=2.16 Myr), when the fault is about to switch to the other side of the ridge axis (see accumulated plastic strain outlining the faults).

S2: Figure of 3-D model with M=0.5

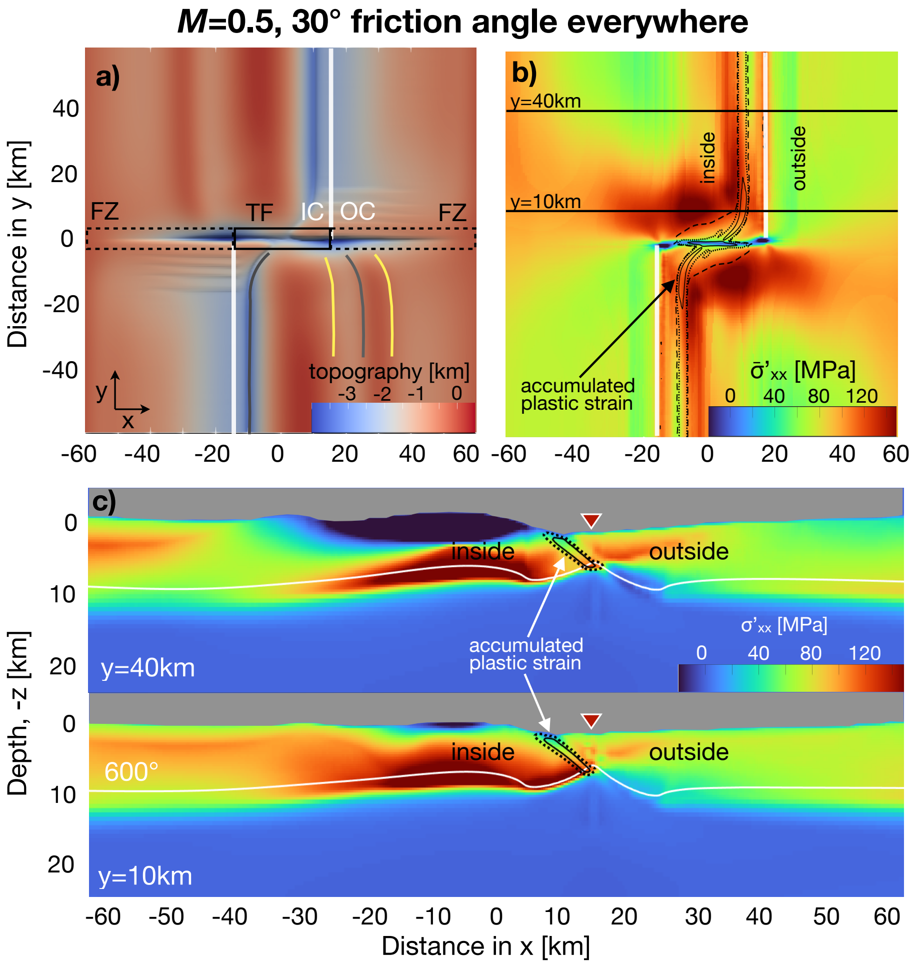

Supplementary Figure S 2. Model results for M=0.5 are shown. For visualization, the images are restricted to +/-60 km in x and y. (a) Map of model topography at 4.0 Myr showing long-lived detachment fault on the IC after faulting switched sides twice. Breakaway (yellow lines) and termination (dark grey lines) for faults on the IC on the southern plate are shown. Dashed dark grey line outlines the active fault. (b) Plan view of deviatoric horizontal normal stress (σ'_{xx}, positive for tension) averaged in depth between the seafloor and the 600° C-isotherm. White lines mark dike injection zones. Black solid and black dotted contours show accumulated plastic strain of 0.2 and 1.2, respectively, revealing faulting on the inside (TF side). Overall, the averaged σ'_{xx} is elevated around the TF, and rather low on the outside all along the ridge segments. (c) Vertical cross-sections of σ'_{xx} at y=40 km and y=10 km (located by black lines in panel (a)). Red triangles locate the dike zone, black contours are as in (a), and white line marks the 600° C-isotherm. Far from the TF (y=40 km, top), downward bending of the hanging wall lithosphere causes σ'_{xx} to be tensile near the top and compressive near the bottom of the lithosphere. Close to the TF (y=10 km, bottom), σ'_{xx} is generally more tensile in the deeper part of the lithosphere attached to the footwall than in the hanging wall.

S3: Calculation of Integrated Strength of Transform Fault and Fracture Zone

In order to explore solutions for a range of friction angles along the transform fault (TF) (φ_{TF}) and fracture zone (FZ) (φ_{FZ}) we consider the relative strengths of the TF (S_{TF}) and FZ (S_{FZ}). The results in the main text are shown in terms of shear stress, integrated over the approximate thickness of the lithosphere (H_l=7 km) and assuming that the stress normal to the fault plane is lithostatic. These calculations take into account that the active TF has lost cohesion (C_{TF}=2 MPa), whereas the FZ has the full cohesion C_{FZ} of 40 MPa. In the strength calculations g=9.8 m/s is used as gravitational acceleration, ρ=3300 kg/m^3 as the density of the lithosphere, ρ_w=1000 kg/m^3 as the density of the water (water column height, H_w=4 km) and μ_{TF} and μ_{FZ} denote the friction coefficient for the TF and FZ, respectively,

$$\begin{aligned} S_{TF} &= \int_{-H_l}^{0} (\mu_{TF}\rho g z + \mu_{TF}\rho_w g H_w + C_{TF}) dz \\ &= \frac{\mu_{TF}\rho g H_l^2}{2} + \mu_{TF}\rho_w g H_w H_l + C_{TF} H_l \end{aligned} \tag{1}$$

$$\begin{aligned} S_{FZ} &= \int_{-H_l}^{0} (\mu_{FZ}\rho g z + \mu_{FZ}\rho_w g H_w + C_{FZ}) dz \\ &= \frac{\mu_{FZ}\rho g H_l^2}{2} + \mu_{FZ}\rho_w g H_w H_l + C_{FZ} H_l. \end{aligned} \tag{2}$$

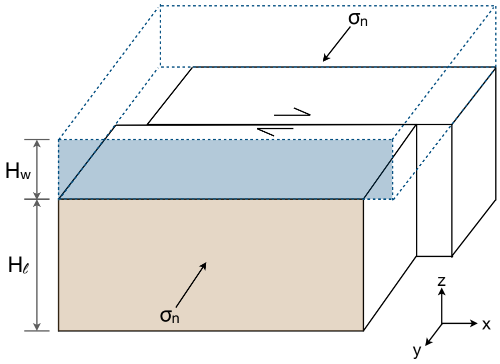

Supplementary Figure S 3. Sketch showing the assumptions behind the strength calculation described in Text S3. H_w is the water column height and H_l the thickness of the lithosphere. σ_n is the normal stress

S4: Movie of Model with tapering M from 0.8-0.6

The movie shows the evolution of the model shown in figure 5 in the main paper. Topography overlies the viscosity field. Black lines outline second invariant of strain rate for $> 10^{-13}$. In the movie it can be seen that faulting switches sides of the ridge axis and that the faults are always marked by high strain rate and low viscosity. The movie can be found at `https://bitbucket.org/JanaNa/lamem/downloads/S4_TFshear_detachmentFaults.mov`.

S5: Movie of Model with $M = 0.6$ and weakened transform fault and fracture zones

The movie shows the evolution of toppography (map view) and two cross sections (of the model shown in figure 3d)-f) in the main paper. In the movie it can be seen that faulting switches sides of the ridge axis initially and that the faulting then localizes on the outside (fracture zone-side) of the ridge axis along both, the northern and southern ridge. Faults are outlined by showing the accumulated plastic strain (0.18). The cross sections are colored according to σ'_{xx}. The movie can be found at `https://bitbucket.org/JanaNa/lamem/downloads/S5_M06_FZ3TF3.mov`.

S6: Movie of Model with $M = 0.6$ without weakened transform fault and fracture zones

The movie shows the evolution of toppography (map view) and two cross sections (of the model shown in figure 3a)-c) in the main paper. In the movie it can be seen that faulting initially switches sides of the ridge axis and that the faulting then localizes on the inside (transform fault-side) of the ridge axis along both, the northern and southern ridge. Faults are outlined by showing the accumulated plastic strain (0.23). The cross sections are colored according to σ'_{xx}. The movie can be found at `https://bitbucket.org/JanaNa/lamem/downloads/S6_M06_30DegreeFrictionAngle.mov`.

S7: Inside or outside corner-detachment faults? - Results of longer transform faults

	30km long (paper)	40km long	50km long
friction angle TF/FZ: 3°/3°	outside	outside	outside
friction angle TF/FZ: 3°/5°	inside	inside	inside
friction angle TF/FZ: 5°/3°	outside	inside	inside
